# A Cross-Sectional Study on the Impacts of Perceived Job Value, Job Maintenance, and Social Support on Burnout among Long-Term Care Staff in Hawaii

**DOI:** 10.3390/ijerph18020476

**Published:** 2021-01-08

**Authors:** Bum Jung Kim, Sun-young Lee

**Affiliations:** 1 Department Social Welfare, Chung-Ang University, Seoul 06911, Korea; swl2001@cau.ac.kr; 2 Department Human Welfare, Prefectural University of Hiroshima, Hiroshima 723-0053, Japan

**Keywords:** burnout, job value, job maintenance, social support, care worker

## Abstract

Extensive research has demonstrated the factors that influence burnout among social service employees, yet few studies have explored burnout among long-term care staff in Hawaii. This study aimed to examine the impact of job value, job maintenance, and social support on burnout of staff in long-term care settings in Hawaii, USA. This cross-sectional study included 170 long-term care staff, aged 20 to 75 years, in Hawaii. Hierarchical regression was employed to explore the relationships between the key independent variables and burnout. The results indicate that staff with a higher level of perceived job value, those who expressed a willingness to continue working in the same job, and those with strong social support from supervisors or peers are less likely to experience burnout. Interventions aimed at decreasing the level of burnout among long-term care staff in Hawaii may be more effective through culturally tailored programs aimed to increase the levels of job value, job maintenance, and social support.

## 1. Introduction

With rapid population aging, the importance of long-term care is growing in most countries affiliated with the Organization for Economic Co-operation and Development (OECD). In 2005, long-term care expenditures accounted for slightly more than 1% of the GDP of all OECD countries, but this number is expected to reach between 2% and 4% by 2050 [1]. Interest in long-term care is expected to grow even more. On the contrary, long-term care is labor-intensive, and its burden is increasing; thus, attracting long-term care staff is becoming more difficult. To respond to the increasing demand for long-term care, investment in policies to utilize the available labor force more efficiently is essential. In particular, the importance of non-financial benefits has recently emerged [2].

The shortage of long-term care staff is one problem occurring in many countries. In particular, the field’s high turnover rate is often pointed out as a cause of the shortage of staff, while job dissatisfaction and burnout are identified as predictors of turnover among long-term care staff [3]. 

The older population in the US, including Hawaii, has been growing rapidly. Between 2015 and 2018, the proportion of the state’s population aged 65 years and older increased from 22.6% to 24.2%, and the percentage of individuals aged 85 and older increased from 2.7% to 3.2% [4]. Given these demographic trends, the demand for long-term care services in Hawaii is expected to grow exponentially over the next few years.

Older people often experience chronically complex health conditions that require long-term treatment. With the increased demand for long-term care derived from the longer average life expectancy and aging population, the workload of staff is increasing. At the same time, the mental stress experienced by staff has attracted attention, and studies from multiple fields have been conducted to identify ways of reducing their mental burden. Therefore, this study examined the factors affecting burnout to develop practical measures for increasing job satisfaction and lowering turnover rates among staff in Hawaii.

### 1.1. Burnout

Burnout refers to the process in which energy related to a job is drained and leads to feelings of helplessness and cynicism in an individual; it largely comprises the sub-concepts of emotional burnout, depersonalization, and lack of personal achievement [5]. The term burnout refers to these and other aspects of job-related stress, which were identified in the 1970s among volunteers of the American Mental Health Center who had lost their motivation to care adequately for clients [6]. In particular, burnout is a negative phenomenon frequently seen in human service professionals, such as doctors, nurses, teachers, counselors, and social workers [7]. 

If burnout persists, it negatively affects physical and psychological well-being. For example, burnout causes disorders like anxiety and depression, decreases job satisfaction and job commitment, diminishes work motivation and productivity, and increases turnover and retirement intentions [8]. Additionally, individuals with a lack of emotional empathy due to burnout might be more inclined to tolerate the abuse of those in their care [9]. 

As mentioned earlier, research suggests that people who provide care services are more likely to experience burnout. Additionally, as the demand for care increases alongside the aging population, researchers’ interest in exploring burnout issues has become more urgent. Maslach et al. [5] classified the factors related to burnout into personal and situational factors. Personal factors include demographic characteristics, personality, and work attitude. Situational factors are classified into job-related characteristics, occupational-related characteristics, and organizational characteristics. Many burnout-related studies have utilized these factors originally identified by Maslach et al. [5]. 

In summary, if burnout persists for an extended period among those who provide care services, they might experience exhaustion and a decrease in motivation to work, which might deteriorate the quality of the services provided and result in abusive behavior toward patients. Therefore, to develop practical support measures for reducing the burnout of staff, we examined factors affecting their burnout.

### 1.2. Theoretical Background 

Among the research models that hypothesize the cause of burnout, the job demands–resources model can be applied as a theory that provides major implications for this study. This theory was proposed by Demerouti et al. [10] as a model of job burnout, applicable to various occupation groups, and was based on the theory of resource conservation [11]. Job resources, referred to herein, are all job-contextual functions that effectively respond to job demands required by the organization, contribute to reducing negative effects (such as job-related stress), and ultimately play a functional role in achieving job goals [10]. For example, they may include a number of individual job-related factors, such as participation in the decision-making process related to the job, diversification of job-related skills, and feedback on the degree of autonomy in performance, along with interpersonal factors, such as cooperative relationships with colleagues and supervisors, and organizational atmosphere [12].

Based on the job demands–resources model, it is assumed that as a variable that affects employees’ burnout, employees’ attitude toward their job—such as high job value, willingness to maintain their job and their relationship with co-workers—is an influential factor [13]. When an organization member experiences a depletion or shortage of job resources, they experience exhaustion due to a decrease in job performance and personal motivation toward achievement.

The direct influences of job attitudes and circumstances (namely, job value and social support) on burnout are well known. There are several studies that show that as the years of service increase, burnout decreases; however, there is a scarcity of studies exploring the inverse causal relationship, which establishes that a decrease in the number of years of service may lead to increased burnout [12,13].

As mentioned above, excessive job factors induce stress in staff; furthermore, they manifest as burnout, which consequently can negatively affect organizational effectiveness [14]. In particular, this study is focused on the existing job demands–resources model, but expands the scope to factors related to favorable work performance environments such as social support and internal motivations such as job value and service providers. According to the results of previous studies, even if job factors negatively impact staff, burnout is likely to decrease when social support is high; internal motivations such as job value and job maintenance are also likely to reduce burnout. Therefore, our analysis could contribute to broadening the scope of application of the job demands–resources model and thereby contribute toward finding ways to reduce burnout.

### 1.3. Literature Review 

#### 1.3.1. Job Value and Burnout

Job value and burnout generally have an inverse relationship: individuals who have a positive emotional outlook regarding their jobs tend to experience a lower level of burnout. For example, in a study on social workers, the higher the value that social workers assigned to their jobs, the lower their levels of burnout were [15]. Similarly, a higher level of burnout was associated with a lack of occupational identity among occupational therapists [16]. Among staff, higher job values have been observed to contribute to a reduction in burnout [17]. In addition, high vocational awareness has been associated with a lower level of burnout among employees in other service sectors [18].

The aforementioned studies suggest that the level of burnout decreases as human service professionals perceive their jobs more positively. Several factors contribute to the positive perception of one’s job, including social reputation and related benefits. Nevertheless, there is a lack of research that has analyzed the effects of individual attributes of job perception on burnout. Therefore, this study focused on and examined the perception of Hawaiian long-term care staff of the social value of their job and its various attributes.

#### 1.3.2. Job Maintenance and Burnout

In a study that examined the relationship between job maintenance and burnout, poor work environment factors, such as long working hours and a lack of regular rest hours, increased burnout among welfare facility workers [14]. Similarly, another study found that job stress and job satisfaction had a significant effect on burnout among nurses [19]. Yet, another study showed a significant relationship between job satisfaction and burnout among counselors. Additionally, many human service professionals continue to work in their respective jobs despite their burnout potential, which provides additional evidence of a relationship between job satisfaction and burnout [20]. Conversely, if job satisfaction decreases and the level of attachment and immersion also declines, workers’ willingness to continue working in the same job also decreases [21]. Therefore, research indicating that as job satisfaction decreases burnout increases, highlights the necessity of examining the long-term impact of the human service profession on workers’ burnout. Therefore, this study also aimed to verify the impact of being a long-term care provider, on burnout.

#### 1.3.3. Social Support and Burnout 

All resources that satisfy one’s physical, material, and emotional needs can be collectively referred to as social support, which reduces the harmful effects of stress, as well as psychological and social burdens [22,23]. Social support also plays a positive role in reducing the burnout of workers by helping them cope with stressful situations [24,25]. Research indicates that social support is a major factor that alleviates burnout by acting as a buffer between stress factors and tension [19,26]. One of the most important forms of social support that individuals have is their network of relationships with others. Studies conducted with social workers indicate that sufficient social support reduces burnout [27,28,29].

Prior studies have demonstrated that social support affects individuals’ experiences of burnout. Additionally, the level of burnout varies among care providers who undergo the same level of stress. Therefore, this study examined the association between different factors (such as job-related social support and the long-term will of caregivers) and burnout, as this notion has not yet been adequately explored. 

#### 1.3.4. Purpose of the Study and Hypotheses

**Figure 1:**
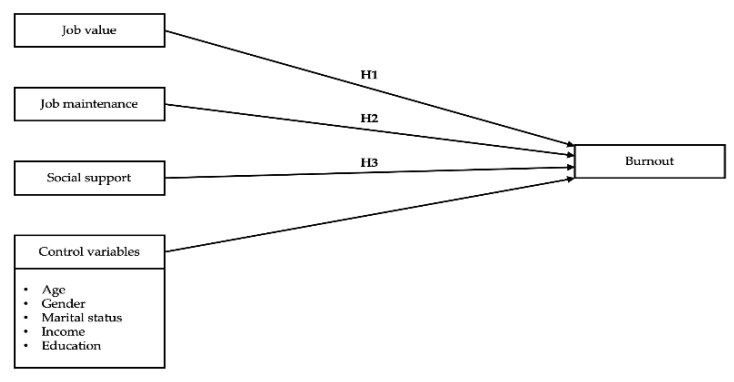
The hypothesized model.

This study aims to examine the relationships between job value, job maintenance, social support, and burnout among long-term care staff in Hawaii, USA. The hypotheses (Figure 1) for the current study are as follows.

**Hypothesis 1** **(H1).** *Job value is correlated with burnout among long-term care staff in Hawaii.*

**Hypothesis 2** **(H2).** *Job maintenance has an influence on burnout among long-term care staff in Hawaii.*

**Hypothesis 3** **(H3).** *Social support is associated with burnout among long-term care staff in Hawaii.*

## 2. Method

### 2.1. Design, Study Site, and Participants 

The study used a cross-sectional survey design with data collected from a convenience sample of 192 long-term care staff in Hawaii, US. The study sample was recruited from 23 long-term care agencies such as nursing homes, adult day care centers, and independent living facilities. Participants in the study were professionals working in long-term care facilities, including nurses, nursing assistants, social workers, physical therapists, and occupational therapists. Staff working at long-term care institutions in Hawaii include social workers, physical therapists, and occupational therapists, as well as nurses and nursing assistants. Social workers play a role in social care and case management, and therapists also play an important role in rehabilitation and dementia management at long-term care institutions. The research team first contacted program directors of long-term care centers and explained the purpose and procedure of the study to obtain their consent for participation. Once agency-wide consent was obtained, the authors identified potential research participants at each agency, obtained informed consent, and distributed self-administered questionnaires for completion. Of the 192 responses obtained, the data from 170 questionnaires were used (22 questionnaires were discarded due to missing data and incomplete responses), representing an acceptance rate of 88.5%. Each participant received USD 5 as compensation.

### 2.2. Data Measures

#### 2.2.1. Burnout

To measure burnout, the study used the Maslach Burnout Inventory (MBI). The MBI was developed by Maslach and Jackson [30]; it comprises three domains (depersonalization, attainment of personal fulfillment, and emotional exhaustion) and 17 items. Participants answered items using a 5-point Likert scale (1 = *almost always,* 2 = *sometimes,* 3 = *every once in a while,* 4 = *rarely,* and 5 = *never*). High scores indicated a high risk of burnout. The only survey item that required inverted calculation due to inverted response values was the attainment of personal fulfillment. The internal consistency reliability (Cronbach’s alpha) for the MBI in this study was 0.91. In addition, confirmatory factor analysis (CFA) was conducted to verify the factor structure of a set of observed items. The CFA produced a chi-square of 5.61 (*p* = 0.47), a root mean square error of approximation (RMSEA) of 0.03, a comparative fit index (CFI) of 0.91, and a Tucker-Lewis index (TLI) of 0.92, all indicating the measure’s reasonably good fit.

#### 2.2.2. Job Value

To measure the level of job value, we used the following item: “How much do you think your current job is valued by society?” Participants responded to the item on a 4-point Likert scale (*very much, somewhat, not much,* and *not at all*). A low score indicated that the participant perceived his or her job as highly valuable.

#### 2.2.3. Job Maintenance

To measure the possibility of participants maintaining their current job, we utilized the following item: “How long would you like to stay in your current workplace?” Participants responded to the item on a 4-point Likert scale (*I would like to quit my job right now, I would like to quit my job but not now, I would like to stay here for the time being,* and *I would like to stay here as long as possible*). A high score indicated a high level of job maintenance.

#### 2.2.4. Social Support

Social support was measured using the Social Support Measurement Tool by Poulin and Walter [31]. This 18-item scale contains five items to measure instrumental support from supervisors, six items to measure emotional support from supervisors, and seven items to measure emotional support from peers. Participants responded to the items using a 4-point Likert scale (*very agreeable, agreeable, almost not agreeable,* and *not agreeable*). A higher score meant stronger social support. In this study, the internal consistency reliability (Cronbach’s alpha) for social support was 0.89. Moreover, CFA analysis produced a chi-square of 7.24 (*p* = 0.23), an RMSEA of 0.01, a CFI of 0.94, and a TLI of 0.97, all indicating the variable’s good fit.

#### 2.2.5. Background Information

Sociodemographic variables were included in this study as follows: age (in years), gender (1 = female), marital status (1 = married), income (continuous variable), and education (continuous variable). 

This study addressed the issue of common method bias. Usually, the concern is that when the same method is used to measure multiple constructs, it may result in spurious method-specific variance that can bias observed relationships between the measured constructs [32]. In order to reduce common method bias, this study used two methods. By adding a time delay, thereby increasing temporality of the items, the study could reduce participants’ tendency to use previous answers to inform subsequent answers. In addition, ambiguous items increase participants’ reliance on their systematic response tendencies as they are unable to rely on the content of the ambiguous item [33]. The study reduced ambiguity by keeping questions as simple and specific as possible.

### 2.3. Ethical Considerations

The authors clearly informed potential participants that their participation was voluntary, that the study adhered to a rigorous protocol for research ethics (guaranteeing participants’ anonymity and confidentiality), and that collected data were to be used for research purposes only. The study design was approved by the Institutional Review Board of the University of Hawaii (CHS #22473) on 9 October 2014.

### 2.4. Data Analysis

There were three procedures for data analysis. First, the study used descriptive statistics to explain the main study variables in terms of frequencies, percentages, and means. Next, the study used bivariate analysis (Pearson’s correlation) to examine the correlational relationships between the independent variables and the dependent variable. Finally, the study performed a robust hierarchical regression analysis with the outlier down-weighting algorithm using STATA version 13.0 software. Four sets of independent variables were regressed on burnout in successive order as follows: (1) sociodemographic characteristics, (2) job value, (3) job maintenance, and (4) social support. Additionally, variance inflation factors were assessed to determine multicollinearity.

To investigate burnout, we first added the sociodemographic variables as a group (age, gender, marital status, education, and income) to control how these factors affect the dependent variable (burnout). Through a hierarchical regression analysis, the change in the R-squared value at each step provided insight into the predictive power of each cluster while controlling the variables in the previous model. To overcome this problem, a robust regression procedure was used to repeatedly reduce or correct outliers. 

## 3. Results

### 3.1. Sample Characteristics

Demographic characteristics of the sample and descriptive data of the following study variables are shown in Table 1: age (range, mean age), gender ratio, marital status ratio, educational level spectrum, average monthly income, the mean scores of job value, job maintenance, social support, and burnout level.

### 3.2. Bivariate Correlations with Burnout 

Imputed correlations between variables from the predicted model are shown in Table 2. Since no correlation coefficient values were over 0.06, multicollinearity was considered nonexistent among the study variables [34]. There was a significant positive correlation between job value (*r* = 0.38, *p* < 0.01) and burnout, indicating that increased negative job value was related to higher levels of burnout. Moreover, there were significant negative correlations between job maintenance (*r* = −0.49, *p* < 0.01), social support (*r* = −0.33, *p* < 0.01), and burnout, indicating that a longer intended stay in the current job and increased levels of social support were associated with lower levels of burnout.

### 3.3. Hierarchical Regression

A hierarchical regression analysis was conducted to examine the impact of job value, job maintenance, and social support on burnout, controlling for the selected demographic variables. Table 3 displays the results of the analysis for the four models. Model 1 included participants’ age, gender, marital status, education, and income; it explained 4% of the total variation in burnout. Of the five demographic predictors, only gender had a significant relationship with burnout (*p* < 0.05). Job value, included in Model 2, explained 18% of the total variation and was positively associated with the level of burnout (*p* < 0.01). Job maintenance was added in Model 3 and explained 35% of the total variance in burnout (*p* < 0.01). Finally, by adding social support to Model 4, it explained 39% of the total variance in burnout. Interestingly, in Model 3, education was a significant variable at the *p*-level < 0.05; however, it was not significant in Model 4. In Model 4, the effect of social support was large, therefore the influence of education was reduced. It can be seen that the level of burnout changes depending on the degree of social support regardless of the educational background. In summary, the results indicated that perceived job value was positively associated with burnout, whereas both job maintenance and social support were negatively associated with burnout.

## 4. Discussion

This study examined the association between burnout and job value, job maintenance, and social support among 170 long-term care staff in Hawaii, US. Several implications for clinicians are provided below based on the major findings of the study.

This study found that job value was negatively associated with burnout among long-term care staff, which supports our first hypothesis. In other words, care staff with a higher perception of job value are more likely to experience a low level of burnout. This is consistent with the findings of earlier research [15,16,17,18]. Long-term care service jobs are often seen as undesirable and difficult, characterized by long work hours, requiring minimal skills, providing low wages, and having high labor intensity [35]. As a result, treatment for long-term care staff tends to be poor, despite the considerable physical, mental, and emotional burden associated with their job, while financial compensation is commensurate with that of a low-quality job. If long-term care staff feel they are not appreciated, their job satisfaction and pride in their work decrease, while stress and burnout are likely to increase.

Efforts are required at the individual, institutional, and government/social levels to increase the extent to which staff value their jobs in long-term care settings. First, staff themselves must recognize that their work helps maintain the human dignity of older adults and the socially disadvantaged. It should be recognized that their assistance as official caregivers does not only increase the independence and life satisfaction of older clients, but also eases families’ care burden and helps family caregivers maintain their social life. Second, it is necessary to provide education and training programs for the professional development of long-term care staff at the institutional level. Specifically, staff should be provided with job training opportunities that require complex skills (such as body care, physical therapy, and rehabilitation), thereby enabling care work to gain acceptance as a viable career choice. In particular, there is a need to expand specialized education on dementia in line with the increasing number of patients with dementia [27]. Additionally, wages and treatment of workers should also be improved to mitigate the negative image of care work. Third, support for long-term care staff should be strengthened at the governmental and societal level. An institutional mechanism is needed to increase the budget support of the central government and to develop policies to improve the treatment of workers by local governments. In particular, it is necessary to actively consider the introduction of public long-term care insurance and to make efforts to facilitate the care of older adults from a public policy standpoint.

This study found a significant negative relationship between job maintenance and burnout among long-term care staff, confirming our second hypothesis. In other words, staff who expressed a willingness to continue working in the same job are more likely to have a low level of burnout. This finding is consistent with earlier research [14,19,20,21]. Having a willingness to work longer means that you are proud and satisfied with your work, and that you are rewarded for your job. Research generally shows that the more employees express a willingness to change their job, the more likely it is that they are unsatisfied with their current job, experience higher levels of stress, and have a higher level of burnout [36]. Thus, it is necessary to improve the work environment, foster the organizational culture of the organization, and adjust the work intensity so staff can stay at their jobs in the long-term.

Additionally, our study revealed that social support was significantly correlated with burnout among staff at long-term care agencies, thus supporting our third hypothesis. Specifically, staff with strong social support from supervisors or peers are less likely to experience burnout; this is consistent with earlier research [19,22,23,24,25,26,27,28,29]. 

The job demands–resources model posits that burnout is influenced by individual job-related factors, such as participation in the decision-making process related to the job, diversification of job-related skills, and feedback on the degree of autonomous performance, but also interpersonal factors, such as social support and cooperative relationships with colleagues and supervisors [12]. Our findings are in concordance with the aforementioned model.

Long-term care staff play a role in helping older people who experience difficulties with activities of daily living as well as instrumental activities of daily living during working hours. Due to the deterioration in physical functioning and cognitive ability of older adults, it is sometimes difficult to communicate with them; consequently, job stress in staff is higher in such cases. In this situation, if the relationship between staff and their supervisor is not productive, the workers’ level of satisfaction with their work will be lower. Conversely, if staff communicate frequently with their supervisors and receive emotional support from them, their mental stress will be reduced. Additionally, if a supervisor provides good supervision and allows staff to discuss their job-related difficulties, the care worker will have a more organized work environment. If relationships with colleagues involve understanding and listening to each other, and staff receive emotional support in a caring attitude, their work life will be easier and more productive. Likewise, staff who receive social support from their supervisor or colleagues will have a lower level of burnout resulting from reduced job stress. In addition, to strengthen the social support for staff, a line of dialogue must be established between staff and supervisors, to allow them the opportunity to communicate openly with each other through meetings and employee training, thereby creating an open organizational culture for solving problems.

This study has a few limitations. First, the study used cross-sectional survey data, which limits our ability to identify causality and time order. Accordingly, future research needs to examine causal relationships among the variables because social support, job value, and job maintenance are time-varying variables, which means they may improve or deteriorate over time. Second, because the participants were recruited from territories in Hawaii from various long-term care facilities (based on non-probability convenience sampling), the findings cannot be generalized to other contexts. Future studies need to recruit study participants from various geographic locations and broaden the applicability of the survey. Third, it is necessary to diversify the questions used in the survey. For example, job value was measured using only one question. Therefore, it is necessary to further subdivide the construct and measure job value as is perceived by the staff themselves and by their family members and acquaintances. Additionally, consideration should be given to including other variables that affect burnout (e.g., physical health, mental health, and job satisfaction) so that it is possible to provide practical policy implications for dealing with burnout by addressing a wider range of influencing factors. Finally, the study did not examine the group differences among staff (e.g., nurses, nursing assistants, social workers, physical therapists, occupational therapists) because the majority of study participants in this study were nurses or nursing assistants and other staff groups are quite small to carry out comparisons. For future study, it is recommended to include an extended number of staff besides the nursing workers. 

## 5. Conclusions

The present study contributes toward an understanding of the effect of job value, job maintenance, and social support on burnout by examining the understudied group of long-term care staff in Hawaii. The higher the value of a worker’s job, the more rewarding and positive it will be. Moreover, as workers remain longer in their position, the relationship with their clients will deepen and close relationships will be formed. Additionally, mutual support between workers and supervisors will increase job satisfaction and reduce work stress, which will eventually improve the quality of services provided to clients. 

Even though previous studies indicate that job value, job maintenance, and social support are significantly associated with the level of burnout, there are limited studies on burnout among staff at long-term care settings in Hawaii. In particular, the relationship between burnout and job value, job maintenance, and social support has not been examined in the previous literature. This study fills the gap by emphasizing the significance of a culturally specific approach. Interventions aimed at decreasing the level of burnout among long-term care staff in Hawaii may be more effective if the levels of job value, job maintenance, and social support are increased through culturally tailored programs. It is necessary to develop a community-friendly and emotional value-oriented program that considers the cultural characteristics of Hawaii. In particular, practitioners can help attenuate burnout among long-term care staff by informing the leaders of long-term care facilities about the importance of job value and social support, to ensure improved quality of care.

## Figures and Tables

**Table 1:** Participants’ characteristics.

Characteristic	Descriptive Statistic	%
Age		
Range	20–75	
*M (SD)*	40.18 (12.41)	
Gender (*n*)		
Male	30	17.65
Female	140	82.35
Marital status (*n*)		
Single (unmarried/widowed/divorced)	98	57.65
Married	72	42.35
Education (*n*)		
Elementary	16	9.41
Middle school	18	10.95
High school	15	8.82
College	48	28.24
Graduate school	73	42.94
Monthly income (dollars)		
Range	USD 400– USD 6500	
*M (SD)*	4383.90 (1058.97)	
Job value		
Range	1–3	
*M (SD)*	1.48 (0.66)	
Job maintenance		
Range	1–4	
*M (SD)*	3.33 (0.73)	
Social support		
Range	0–54	
*M (SD)*	41.45 (9.93)	
Burnout		
Range	0–46	
*M (SD)*	17.04 (9.05)	

**Table 2:** Correlations among study variables.

	1	2	3	4	5	6	7	8
1. Age	-							
2. Gender	−0.00							
3. Marital status	−0.24 **	−0.04						
4. Education	0.24 **	0.02	−0.09					
5. Income	0.13	0.04	−0.15 *	0.18 *				
6. Job value	0.01	0.03	−0.05	−0.05	0.07			
7. Job maintenance	0.05	−0.06	0.06	−0.12	−0.04	−0.28 **		
8. Social support	−0.00	−0.04	0.13	0.12	0.11	−0.25 **	0.20 **	
9. Burnout	−0.05	0.13	0.03	−0.12	−0.05	0.38 **	−0.49 **	−0.33 **

* *p* < 0.05. ** *p* < 0.01.

**Table 3:** Standardized coefficients from robust hierarchical regression on burnout.

Variables	Model 1		Model 2		Model 3		Model 4	
	β	*t*	β	*t*	β	*t*	β	*t*
Age	−0.01	−0.19	−0.02	−0.37	0.01	0.24	0.01	0.17
Gender	0.14	2.04 *	0.12	1.92	0.10	1.85	0.10	1.81
Marital status	−0.00	−0.02	0.02	0.28	0.04	0.69	0.07	1.06
Education	−0.13	−1.52	−0.09	−1.10	−0.15	−2.04 *	−0.13	−1.75
Income	−0.04	−0.74	−0.07	−1.05	−0.07	−1.62	−0.04	−1.05
Job value			0.37	4.80 **	0.25	3.78 **	0.20	3.17 **
Job maintenance					−0.43	−5.77 **	−0.39	−4.87 **
Social support							−0.21	−2.74 **
*R* ^2^	0.04		0.18		0.35		0.39	
*R*^2^ change			0.13 **		0.17 **		0.04 **	
Adjusted *R*^2^	0.06		0.21		0.37		0.41	
*F*	1.43 **		5.49 **		10.52 **		10.98 **	

* *p* < 0.05, ** *p* < 0.01.

## Data Availability

The data presented in this study are available on request from the corresponding author. The data are not publicly available due to privacy or ethical reason.
